# Patient-Derived Induced Pluripotent Stem Cells and Organoids for Modeling Alpha Synuclein Propagation in Parkinson's Disease

**DOI:** 10.3389/fncel.2018.00413

**Published:** 2018-11-09

**Authors:** Yong Hui Koh, Li Yi Tan, Shi-Yan Ng

**Affiliations:** ^1^Institute of Molecular and Cell Biology, Singapore, Singapore; ^2^Department of Physiology, Yong Loo Lin School of Medicine, National University of Singapore, Singapore, Singapore; ^3^National Neuroscience Institute, Singapore, Singapore; ^4^The Third Affliated Hospital of Guangzhou Medical University, Guangzhou, China

**Keywords:** iPSCs, alpha synuclein (α-synuclein), lewy body disease, organoids, disease modeling

## Abstract

Parkinson's disease (PD) is an age-associated, progressive neurodegenerative disorder characterized by motor impairment and in some cases cognitive decline. Central to the disease pathogenesis of PD is a small, presynaptic neuronal protein known as alpha synuclein (a-syn), which tends to accumulate and aggregate in PD brains as Lewy bodies or Lewy neurites. Numerous *in vitro* and *in vivo* studies confirm that a-syn aggregates can be propagated from diseased to healthy cells, and it has been suggested that preventing the spread of pathogenic a-syn species can slow PD progression. In this review, we summarize the works of recent literature elucidating mechanisms of a-syn propagation, and discussed the advantages in using patient-derived induced pluripotent stem cells (iPSCs) and/or induced neurons to study a-syn transmission.

## Introduction

Parkinson's disease (PD) is a progressive and chronic neurological disorder and the second most prevalent neurodegenerative disease after Alzheimer's disease, affecting an estimated seven to ten million people worldwide. Dopaminergic (DA) neurons in the substantia nigra par compacta (SNpc) are selectively lost in PD, leading to a constellation of motor deficits that include bradykinesia (slowed movements), tremors and muscle rigidity. In some patients, symptoms of dementia are also present. Approximately 15% of people with PD have a family history of the disease (termed familial PD) while the others are sporadic cases. Mutations in several genes are each associated with the occurrence of PD, including alpha synuclein (SNCA), leucine-rich repeat kinase 2 (LRRK2), and other autosomal recessive mutations in genes such as Parkin (PARK2), PTEN-induced putative kinase 1 (PINK1) and Protein/nucleic acid deglycase DJ-1 (PARK7), and has been extensively reviewed in Klein and Westenberger (2012). Although the exact cause for sporadic PD is yet to be identified, the largest risk factors for PD are genetics, advanced age and exposure to environmental toxins such as paraquat (Ascherio and Schwarzschild, 2016).

One of the pathological hallmarks of PD is the formation of intracellular inclusions termed Lewy bodies, which are caused by aggregates of a-syn protein. Multiple *in vivo* studies—both human and mouse—have confirmed that a-syn aggregates can be transferred from affected neurons to healthy neural cells (Kordower et al., 2008; Li et al., 2008; Pan-Montojo et al., 2012; Recasens et al., 2014). It is becoming increasingly appreciated that misfolded a-syn can transmit to anatomically connected areas (Braak et al., 2003), and this could explain why a substantial proportion of PD patients also suffer from cognitive impairment, depression and psychosis. Several mechanisms of a-syn transmission have been proposed, including receptor-mediated endocytosis, direct cell-to-cell transfer through tunneling nanotubes or through a trans-synaptic pathway (Pan-Montojo et al., 2010; Luk et al., 2012b; Holmes et al., 2013; Abounit et al., 2016; Mao et al., 2016; Rostami et al., 2017). Although the mechanism of spread remains slightly controversial, it is well accepted that limiting the spread of a-syn aggregates can slow the progression of PD, and potentially prevent other PD-associated decline in cognitive functions.

In recent years, scientific advances in the field of induced pluripotent stem cells (iPSCs), direct reprogramming into induced neurons and the formation of neural organoids have enabled the modeling of PD using patient-derived cells, and opened up possibilities for the discovery of prognostic and therapeutic agents. Over the years, differentiation protocols have dramatically evolved to give rise to specific midbrain DA neuron populations that are lost in PD. From co-culture with mouse PA6 or MS5 stromal cells (Kawasaki et al., 2000; Perrier et al., 2004) that gave rise to low DA neuron yield, midbrain DA differentiation has now become more reproducible and efficient with chemically defined protocols (Kriks et al., 2011; Kirkeby et al., 2012; Doi et al., 2014; Paik et al., 2018). Disease modeling efforts by multiple groups worldwide has now uncovered that midbrain DA neurons derived from PD patients exhibit mitochondrial dysfunction and a-syn aggregation (Devi et al., 2008; Byers et al., 2011; Cooper et al., 2012; Imaizumi et al., 2012; Ryan et al., 2013; Flierl et al., 2014; Shaltouki et al., 2015; Chung et al., 2016; Kouroupi et al., 2017). iPSC-derived midbrain DA neurons are also useful for potential cell replacement therapies, an undertaking that is initiated by the GForce-PD group, a global team of scientists and clinicians that are committed to accelerate the translation of stem cell-based therapies to the clinic for Parkinson's disease human trials (Barker et al., 2015). While cell replacement therapy can correct the motor deficits in PD patients, it is unlikely to rectify the non-motor symptoms such as dementia, depression, delusions or hallucinations, which are common in advanced-staged PD patients. Therefore, slowing down PD progression remains an attractive therapeutic option. The focus of this mini-review will be to highlight the complexity of a-syn propagation and how iPSC-derived cell types and organoids can address some of this complexity.

## Alpha synuclein propagation as the central mechanism in the development of PD

Lewy bodies and lewy neurites are the histological hallmark of PD. The main protein constituent of Lewy bodies and Lewy neurites is a-syn, a 140-amino acid presynaptic nerve terminal protein that comprises an amphipathic N-terminal alpha-helical domain, a hydrophobic center of non-amyloid beta component and a hydrophilic C-terminal domain. Under the native physiological state, a-syn does not have a defined structure and exists in an amorphic state. Although the exact functions of a-syn is still unknown, knockout studies have revealed roles of a-syn in synaptic vesicle release and trafficking, fatty acid binding, and the regulation of enzymes and transporters that are essential for neuronal survival (Sharon et al., 2001; Kanaan and Manfredsson, 2012; Stefanis, 2012). In the pathological state, a-syn becomes misfolded and therefore prone to aggregation. First, it forms soluble oligomers and then further aggregate into insoluble fibrils. These insoluble fibrils are made up of β-sheets consisting of two or more polypeptide chains connected by hydrogen bonds. Although the exact pathogenic form of a-syn is still debatable, recent studies suggest that soluble oligomers could be more toxic than insoluble fibrils (Karpinar et al., 2009; Winner et al., 2011); presumably because soluble oligomers can be transmitted more readily than insoluble fibrils. The misfolding, aggregation and accumulation of a-syn has serious neurotoxic implications (Stefanis et al., 2001; Tanaka et al., 2001; Snyder et al., 2003; Cuervo et al., 2004; Xilouri et al., 2009; Kamp et al., 2010; Nakamura et al., 2011), and is extensively reviewed in Lashuel et al. (2012). A-syn is also thought to be the pathogenic agent that underlies the progression of PD when toxic a-syn species transmit from diseased to healthy cells.

Braak and colleagues first suggested a prion-like mechanism in PD progression (Braak et al., 2003). They proposed that Lewy pathology spread through a stereotyped pattern of six stages, beginning from the peripheral nervous system of the gut and olfactory bulb and gradually progresses into the central nervous system. Within the brain, it spreads from the brainstem to the multiple cortical regions of the brain in a caudal-to-rostral fashion. Following on, in 2008, the serendipitous discoveries from two separate studies uncovered the presence of Lewy bodies in grafted neurons of PD patients whom have received transplantation a decade or two ago (Kordower et al., 2008; Li et al., 2008). Such observation further supports the prion-like spreading of a-syn in PD. Subsequently, many groups have attempted to recapitulate the prion-like capacity of a-syn in *in vivo* and *in vitro* models. The first few studies demonstrated the host-to-graft transfer of a-syn by transplanting neural stem cells or naïve rodent neurons into the brains of transgenic mice expressing human a-syn (Desplats et al., 2009; Hansen et al., 2011; Kordower et al., 2011; Angot et al., 2012). It was shown that human a-syn was taken up by the grafted cells and can act as a seeding template or a nucleation process to form aggregates with the intracellular mouse a-syn. In a unique mouse model that incorporates both preformed fibrils (PFFs) and a-syn overexpression, it was also shown that PFFs were necessary for the transmission of a-syn as simply overexpression of a-syn was not sufficient to result in the propagation phenomenon (Thakur et al., 2017). Other studies went even further to prove the prion-like capacity of a-syn when pathogenic a-syn were detected in neurons distant from the site of injection (Luk et al., 2012a,b; Mougenot et al., 2012; Rey et al., 2013; Sacino et al., 2013; Recasens et al., 2014; Peelaerts et al., 2015; Shimozawa et al., 2017).

The molecular mechanisms of a-syn propagation are slowly becoming unraveled. Detection of extracellular a-syn confirmed that cells secrete a-syn either as a naked entity or packaged into exosomes and exocytosed (Emmanouilidou et al., 2010; Danzer et al., 2012). Soluble oligomeric and monomeric a-syn were readily detected in cell culture media, in a calcium-dependent manner (Emmanouilidou et al., 2010), suggesting that dysregulation in neuronal activity can impact a-syn exocytosis and propagation. In humans, monomeric and oligomeric a-syn are also detected in human blood plasma and cerebrospinal fluid (Borghi et al., 2000; El-Agnaf et al., 2006; Lee et al., 2006; Tokuda et al., 2006), with increased levels of oligomeric a-syn in PD patients (El-Agnaf et al., 2006). It is also shown that elevated a-syn burden, caused by increased a-syn production (through overexpression or duplication and triplication mutations), or reduced clearance through lysosomal or proteosomal systems, would increase a-syn exocytosis (Alvarez-Erviti et al., 2011; Lee et al., 2013; Fernandes et al., 2016). Extracellular a-syn can interact with different surface proteins on the cells that facilitate its uptake via receptor-mediated endocytosis. Heparan sulfate proteoglycan interact with a-syn fibrils and induce uptake via macropinocytosis (Holmes et al., 2013). By means of a proteomics screen, Mao et al. (2016) has identified a few surface proteins that interact well with preformed fibrils (PFFs) of a-syn (Mao et al., 2016). Lymphocyte-activation gene 3 (LAG3) was one receptor found to have the strongest interaction specifically to a-syn PFFs but not monomers. LAG3, a transmembrane protein, facilitates the uptake of a-syn PFFs in neighboring neurons, astrocytes and microglial via endocytosis. Through genetic knockdown and antibody-blocking intervention, uptake of a-syn was reduced, which led to decreased neuronal toxicity and inter-neuronal propagation *in vitro* and *in vivo* (Mao et al., 2016).

One major caveat of the abovementioned studies is the assumption that fibrillar forms of a-syn are present extracellularly. Indeed, a-syn has been detected in human cerebrospinal fluid (CSF) exosomal vesicles (Alvarez-Erviti et al., 2011; Danzer et al., 2012; Stuendl et al., 2016), but these are mainly soluble a-syn monomers or oligomers. It is not clear whether fibrillar forms of a-syn are present extracellularly.

## Various neural cell types contribute to alpha synuclein pathology

Alpha synuclein deposits are also found in astrocytes at advanced disease stages (Braak et al., 2007) and in oligodendrocytes as glial cytoplasmic inclusions (Ubhi et al., 2011). This means that pathogenic a-syn can also be transferred from neurons to other cell types such as astrocytes and oligodendrocytes. Astrocytes have been observed to take up extracellular a-syn *in vitro* (Lee et al., 2010; Fellner et al., 2013; Rannikko et al., 2015; Lindström et al., 2017). Glial cytoplasmic inclusions develop despite the lack of a-syn mRNA in oligodendrocytes (Miller et al., 2005), suggesting that a-syn is not produced by the oligodendrocytes but rather internalized from the external microenvironment. Studies have shown that monomeric and oligomeric forms in a-syn are internalized by oligodendrocytes *in vitro* (Kisos et al., 2012; Konno et al., 2012) and *in vivo* (Reyes et al., 2014) with suggestions of dynamin-mediated mechanisms involved in the uptake. Overall, the role of astrocytes and oligodendrocytes in pathogenic a-syn propagation or PD disease progression remain largely unknown. Studies by Abounit et al. (2016) proposed a model for propagation of pathogenic a-syn species by interneuronal transfer of fibrillar a-syn-laden lysosomes. a-syn PFFs within specialized cellular structures known as tunneling nanotubes (TNTs) were detected and these can seed soluble a-syn aggregation in the cytosol of recipient cells (Abounit et al., 2016). Apart from interneuronal TNTs, inter-astrocytic TNTs also spread a-syn aggregates. Using human embryonic stem cell-derived astrocytes, Rostami et al. (2017) demonstrated that failure of diseased astrocytes to degrade a-syn PFFs led them to unload their burden to surrounding astrocytes through TNTs (Rostami et al., 2017).

It is postulated that astrocytes and microglia play key roles in clearance of toxic a-syn species from the extracellular environment, and consistent with this notion, astrocytes are capable of extensive uptake of a-syn oligomers, which they then attempt to degrade via the lysosomal pathway (Lindström et al., 2017). Incomplete degradation caused by a-syn overburden can result in cellular damage in recipient astrocytes, including lysosomal defects and mitochondrial damage. Internalization of a-syn has also been shown to cause astrocyte activation and neuroinflammation (Yu et al., 2018), which impacts neuronal survival. Microglia are the primary immune cells of the central nervous system, and are known to be activated by aggregated a-syn (Zhang et al., 2005). In particular, microglial phagocytosis of a-syn was thought to be a mechanism that promotes a-syn clearance. It has been reported by several studies that toll-like receptor 4 (TLR4) is required for a-syn dependent activation of microglia, and TLR4 ablation led to impaired microglia phagocytosis and suppressed cytokine release, enhancing neurodegeneration in those mice (Stefanova et al., 2011; Fellner et al., 2013).

Taken together, these are key evidence supporting the notion that propagation of a-syn is a key driver underlying PD pathogenesis and progression; and that interaction between multiple cell types regulate this process. Therefore, cellular systems using neuroblastoma cell lines (such as mouse Neuro-2a and human SH-SY5Y) or neural stem cell lines do not recapitulate the complexity of a-syn propagation. Animal models are also valuable tools for studying a-syn propagation. To this end, wild-type mice with a single inoculation of a-syn fibrils or pathological a-syn purified from postportem PD brains showed a-syn propagation to anatomically connected brain regions (Luk et al., 2012b; Blesa and Przedborski, 2014; Masuda-Suzukake et al., 2014; Recasens et al., 2014) that is reviewed in Blesa and Przedborski (2014). Though important, the conservation of a-syn transmission between mouse and human has to be established, and that eventual drug screening approaches would be more feasible and have a high throughput if performed in cultured human cells. Therefore, we propose that human induced pluripotent stem cell (iPSC)-derived neurons and neural organoids are ideal cellular platforms for studying a-syn pathology.

## Induced pluripotent stem cells and midbrain differentiation

Induced pluripotent stem cells or iPSCs revolutionize the way human diseases are modeled *in vitro*. iPSCs are typically skin or blood cells genetically reprogrammed to revert back to an embryonic stem cell (ESC)-like state by ectopic expression of ESC transcription factors OCT4, SOX2, c-MYC and KLF4 (Takahashi and Yamanaka, 2006). Several methods of reprogramming iPSCs have now been described (Takahashi and Yamanaka, 2006; Carey et al., 2009; Sommer et al., 2009; Somers et al., 2010) and are also summarized in Table 1 and reviewed in Malik and Rao (2013) and Seki and Fukuda (2015). Importantly, these iPSCs behave like ESCs with the capacity to self-renew and retain its pluripotency. iPSCs also retain the genetic mutations from their donors, making these attractive cellular models for modeling human diseases. Thus far, human iPSCs has become a promising tool to address the ethical issues of handling embryonic material, clinical applications for personalized treatments, and research applications as model systems to investigate human diseases in the fields of neuro-developmental and degenerative diseases (Ardhanareeswaran et al., 2017).

**Table 1:** Gene delivery methods used for iPSC generation.

**Methods**	**Types**	**Subtypes**	**Advantages**	**Disadvantages**
Viral	Integrating	Lentiviral (Somers et al., 2010)	Ability to infect non-dividing and proliferating cells i.e., somatic cells	Incorporation of vector sequence into host genome Solution: single cassette reprogramming vector & cre/loxp mediated transgene excision e.g., STEMCCA
	Non-integrating	Adenovirus (Zhou and Freed, 2009)	Does not integrate into host genome	Very low reprogramming efficiency compared to lentiviral delivery
		Sendai virus (RNA virus) (Fusaki et al., 2009; Ban et al., 2011)	Does not enter nucleus and gets diluted out of cells Can produce large amounts of protein	Difficult to remove replicating virus
Nonviral	mRNA transfection (Warren et al., 2010)		No integration into host genome Higher efficiency than original retroviral system Commercially available	Labor intensive Technically complex
	miRNA transfection (Miyoshi et al., 2011; Subramanyam et al., 2011)		Absence of breaks in existing genes Avoids reactivation of transgenes	No established reprogramming protocol available
	Transposons i.e., Piggybac (Kaji et al., 2009; Woltjen et al., 2009; Yusa et al., 2009)		Highly active in mammalian cells Vector can be removed from the host genome by expressing transposase	Low reprogramming efficiency
	Episomal plasmids (Yu et al., 2009; Chen et al., 2011)		No integration into host genome More stable expression compared to standard plasmids	Requires changes to cell culture methods
	Recombinant proteins (Kim et al., 2009; Zhou et al., 2009)		Absence of breaks in existing genes Avoids reactivation of transgenes	Lower reprogramming efficiency compared to retroviral systems Challenging to generate and purify
	Small molecules (Hou et al., 2013)		Nonimmunogenic Easy to handle	No established protocol for human somatic cells

For meaningful disease modeling, one of the greatest hurdles is to be able to produce large amounts of the cell type of interest with high efficiency and reproducibility. One of the earliest methods of deriving DA neurons from ESCs was to co-culture with stromal feeder cells MS5 or PA6 (Kawasaki et al., 2000; Perrier et al., 2004). This stromal co-culture method, however, was chemically undefined, resulting in a heterogeneous population of neurons with overall low DA neuron yield, and the physical co-culture of human iPSCs with mouse stromal cells made it undesirable for downstream analyses or applications. The labs of Lorenz Studer and Malin Parmar have made significant progress in a chemically defined protocol for efficient differentiation of midbrain DA neurons. These methods made use of the knowledge on developmental patterning to efficiently differentiate iPSCs into midbrain-regionalized floorplate progenitor cells (Fasano et al., 2010; Kirkeby and Parmar, 2012) using chemical inhibitors of SMAD signaling (achieved by LDN-193189 and SB431542), early high-dose Sonic Hedgehog (SHH) pathway agonists (such as Purmorphamine or recombinant SHH) and partial glycogen synthase kinase (GSK) inhibitors/Wnt activation (by CHIR99021) (Cooper et al., 2010; Devine et al., 2011; Kriks et al., 2011; Kirkeby et al., 2012). This revised strategy produces midbrain DA neurons that expresses the specific forehead box protein A2 (FOXA2) and LIM Homeobox Transcription Factor 1 Alpha (LMX1A) markers and demonstrates efficient dopamine release *in vitro* (Kirkeby et al., 2012) and *in vivo* after transplantation (Kriks et al., 2011).

However, even with a chemically-defined approach, a heterogeneous mix of both substantia nigra pars compacta (SNpc or A9-subtype) and ventral tegmental area (VTA or A10-subtype) DA neurons are produced, and it remains a challenge to derive only A9 DA neurons—the neuronal subtype that is lost in PD. Previous work from the laboratories of Ole Isacson and Thomas Perlmann showed that the transcription factor Orthodenticle Homeobox 2 (Otx2) is a marker, and controls the specification of mouse A10 VTA DA neurons, while Sox6 defines the A9 SNpc DA neurons (Panman et al., 2014). SOX6 is also shown to localize to neuromelanin and Tyrosine hydroxylase (TH)-positive neurons in the human SNpc (Panman et al., 2014). A recent article that made use of single cell RNA profiling confirmed that Sox6 and Otx2 mark SNpc and VTA neurons respectively, while also adding a panel of genes specific to SNpc vs. VTA that they found from single cell RNA-seq (Poulin et al., 2014). This genetic information would be helpful in subsequent efforts to direct A9 DA neuron-specific differentiation. One possibility is to overexpress SOX6, or knockdown OTX2 expression in iPSC-derived floorplate cells as they differentiate into neurons. It has previously been shown in mice that ablation of Otx2 results in severely diminished VTA DA neuron differentiation (Di Giovannantonio et al., 2013) but it remains to be determined if overexpression of SOX6 and/or knockdown of OTX2 will drive the SNpc DA neuron transcriptional program in human iPSC-derived cultures.

## Brain organoids and disease modeling

More recently, the ability to generate three-dimensional (3D) neural organoids has challenged the way we think about conventional cellular differentiation and disease modeling approaches. The two-dimensional approach to differentiate cells forces cells into a monolayer that is uniformly exposed to extracellular signals but does not represent their *in vivo* context, and does not fully maintain the complex cell-cell and cell-matrix interactions, resulting in the tendency to lose important physiological function. A landmark paper by Lancaster et al. (2013) showed that neural organoids mimick the cytoarchitecture of the developing cortex. The development of a protocol for brain-like organoids focused on two aims: the induction and differentiation of neural tissue and achieving a 3-D spatial organization that captures the development of specific brain regions. Firstly, iPSCs can be stimulated to form germ layers within iPSC aggregates known as embryoid bodies (EBs) (Evans, 2011). Specific media compositions are used to induce the formation of neural rosettes (Zhang et al., 2001) (polarized organization of epithelial cells) within the EBs. The subsequent change to differentiation medium (usually Neurobasal medium and B27 supplement for neuronal survival and differentiation with specific morphogens) facilitates the development of an organized neuroepithelium that would form various brain structures. Due to the absence of a basement membrane for the EBs to establish proper apical-basal polarity to form the neuroepithelium, an external structural support is required to ensure proper orientation of the neuroepithelium. For most organoid protocols, hydrogels (usually matrigel) are used to encapsulate the EBs to promote the accurate growth and formation of brain-like structures. When EBs are encapsulated within stagnant matrigel droplets, the diffusion of nutrients and oxygen is very poor causing death to the cells at the center of the organoids. Hence, after establishing the proper growth and differentiation within the matrigel droplet, the organoids have to be cultured in a spinning bioreactor to increase diffusion efficiency that promotes tissue survival. Neural organoids can capture the key features of the human brain such as ventricle-like spaces, distinct proliferative layers of cells, and the choroid plexus (Marton and Paşca, 2016); offering a great potential to be used as models of neurodevelopmental and neurodegenerative conditions. Furthermore, protocols have already been established for various brain regions such as cerebral (Lancaster and Knoblich, 2014; Muguruma et al., 2015), forebrain (Qian et al., 2016), and midbrain (Jo et al., 2016) organoids.

Although mostly used to model neurodevelopmental diseases, neural organoids can also be extremely useful for modeling a degenerative disorder such as PD. Since organoids mimick the brain's microenvironment, it has been postulated that culturing of neurons in such a 3D microenvironment would promote their maturation. Jo et al. (2016) reported the generation of a midbrain organoid with A9 neurons that produces neuromelanin (a dark pigment expressed in the SNpc of humans). So far, none of the 2D differentiation protocols have given rise *in vitro* to neuromelanin-producing DA neurons. Of significance, the accumulation of neuromelanin in DA neurons increases with age, suggesting that DA neurons in organoids are far more mature than those in 2D. Comparisons of gene expression between DA neurons cultured in 2D vs. 3D organoids also suggest that neurons in organoids are more mature, expressing dopamine transporter (DAT or SLC1A3) (Jo et al., 2016; Monzel et al., 2017), and genetically resembling the prenatal midbrain (Jo et al., 2016). Recently, Monzel et al. (2017) managed to derive midbrain-specific organoids that contained spatially-organized groups of dopaminergic neurons with other neuronal, astroglial, and oligodendrocyte differentiation. Functionally, they detected the presence of synaptic connections and electrophysiological activity. Since PD is an age-onset neurodegenerative disorder, it is critical to model cellular and molecular aspects of the disease with mature and aged neurons rather than neurons of an embryonic resemblance. Moreover, the heterogeneity of cell types within midbrain organoids would be useful to study the interplay and contributions of other cell types to the a-synuclein pathology of PD. As such, midbrain organoids are a very promising platform for investigating late phenotypes associated with PD–a unique feature that 2D culture models cannot offer.

## Direct reprogramming of fibroblasts into induced dopaminergic neurons (iDANs)

Apart from differentiation of iPSCs toward DA neurons that mimic neural developmental processes, overexpression of key transcription factors in patient-derived fibroblasts can be directly transdifferentiated into neurons, including midbrain DA neurons (Xu et al., 2017). Wernig and colleagues have reported viral-based transdifferentiation of mouse and human fibroblasts into induced neurons (iNs) by overexpressing up to four neuronal transcription factors, namely, achaetescute homolog 1 (ASCL1), BRN2 (also known as POU3F2), myelin transcription factor 1-like protein (MYT1L) and neuronal differentiation 1 (NEUROD1) (Vierbuchen et al., 2010; Pang et al., 2011). These iNs obtained were morphologically and electrophysiologically similar to bona fide neurons, and resembled excitatory neurons of the cerebral cortex (Heinrich et al., 2014). Building onto this knowledge of direct reprogramming, it has also been shown that midbrain DA neurons can be directly converted from fibroblasts. To do so, several groups have reported direct reprogramming of DA neurons using a cocktail of transcription factors specific to the midbrain lineage (Table 2). The factors that were used for induced DA neurons (iDANs) are extensively reviewed in Jang and Jung (2017). Overall, regardless of the combination of transcription factors used, the efficiency of iDAN conversion from fibroblasts is typically below 20%, even though iDANs demonstrated spontaneous and rebound action potentials which are characteristics of midbrain DA neurons *in vivo*. This low efficiency of conversion is potentially a limiting factor for disease modeling studies especially when large numbers of cells are required for high throughput screening. Recently, this hurdle has been overcome by a reprogramming strategy that involves ASCL1, LMX1A, and NURR1 in combination with p53-small hairpin RNA (shRNA) and miR-124, as well as small molecule and trophic factor supplements that maintain the identity and survival of midbrain DA neurons (Jiang et al., 2015). This transdifferentiation approach resulted in more than 50% TH^+^ iDANs, and it was concluded from this study that G1 arrest was crucial for efficient transdifferentiation, and that addition of patterning small molecules such as SB431542, CHIR99021, Purmorphamine (SHH pathway agonist), Dorsomorphin and trophic factors significantly enhanced reprogramming efficiency.

**Table 2:** List of different strategies used to derive induced dopaminergic neurons. Adapted and revised from Jang and Jung (2017).

**No**.	**Type of transdifferentiated cells**	**Transcription factors**	**miRNA**	**Small molecules**	**TH+ differentiation efficiency**	**Characterization tests**	**References**
**1**	Human induced dopaminergic neurons (iDAN)	Ascl1, Brn2, Myt1l, Lmx1a and FoxA2	N/A	N/A	~10%	Expression of dopaminergic neuron markers and electrophysiological profile of functional dopaminergic neurons	Pfisterer et al., 2011
**2**	Mouse and human iDAN	Ascl1, Lmx1a and Nurr1	N/A	N/A	~15%-20%	Expression of dopaminergic neuron markers, electrophysiological profile of functional dopaminergic neurons and dopamine release	Caiazzo et al., 2011
**3**	Mouse iDAN	Ascl1, Lmx1b and Nurr1	N/A	N/A	~18%	Expression of dopaminergic neuron markers, electrophysiological profile of functional dopaminergic neurons and dopamine release	Addis et al., 2011
**4**	Mouse iDAN	Ascl1, Pitx3, Lmx1a, Nurr1, FoxA2 and EN1	N/A	Sonic hedgehog (Shh) and fibroblast growth factor 8 (FGF8)	~7%	Expression of dopaminergic neuron markers, electrophysiological profile of functional dopaminergic neurons, dopamine release and relief PD-like symptoms in PD mice	Kim et al., 2011
**5**	Human iDAN	Ascl1, Ngn2, Sox2, Nurr1 and Pitx3	N/A	N/A	~40%	Expression of dopaminergic neuron markers, dopamine uptake and release, electrophysiological profile of functional dopaminergic neurons and relief PD-like symptoms in PD mice	Liu et al., 2012
**6**	Human iDAN	Ascl1, Lmx1a and Nurr1	miR124	p53 suppressor, G1 cell cycle arrest and Tet1 agonist	~60%	Expression of dopaminergic neuron markers, DA uptake and release, electrophysiological profile of functional dopaminergic neurons	Jiang et al., 2015
**7**	Mouse induced neural progenitor cells (iNPCs) with midbrain identity	Foxa2, Brn2 and Sox2	N/A	N/A	~90%	Expression of dopaminergic neuron proliferative progenitor cell markers, capable of deriving functional dopaminergic neurons and to rescue MPTP-lesioned mice	Tian et al., 2015

Another method for generating DA neurons from patient fibroblasts is to derive expandable dopaminergic precursors known as floorplate progenitor cells. This has been achieved in mouse fibroblasts by ectopic expression of Brn2, Sox2, and FoxA2 (Tian et al., 2015), resulting in acquisition of floorplate identity which include expression of Otx2, Corin and Lmx1a expression. Induced floorplate progenitors generated with this method were shown to differentiate primarily into TH^+^ midbrain DA neurons (with more than 90% efficiency), even without addition of Shh and Fgf8. Although this has not been demonstrated for human cells, we expect similar results based on previous iN studies where the same reprogramming factors worked similarly in mouse and human cells. If so, this could be an ideal method for disease modeling because large numbers of DA neurons can be derived from these self-renewing induced floorplate progenitors.

Although transdifferentiation technologies may not be compatible with neural organoid formation, because directed reprogramming forces fibroblasts to take on a specific cell fate rather than allow for a “self-organizing” approach that is important for organoid formation, there are distinct advantages in using iNs for disease modeling. Two recent studies (Mertens et al., 2015; Huh et al., 2016) found that iNs from aged fibroblasts retained the aging cellular and molecular characteristics while iPSCs made from the same patient fibroblasts were epigenetically reprogrammed to erase the aging signatures and subsequent neurons differentiated from these iPSCs did not acquire aging characteristics. Since PD is an age-onset neurodegenerative disease, iNs that retain aged signatures could be an especially relevant cellular model to understand the role of aging in neuronal decline. It is unclear, however, if induced floorplate progenitors retain aged cellular signatures that also make them suitable models for studying aged-associated neuronal decline.

## iPSC-derived midbrain cultures as an *in vitro* model of a-syn transmission

Despite numerous *in vivo* and *in vitro* studies that were discussed in the previous sections demonstrating transmission and propagation of a-syn in PD pathology, there is still a lack of a robust and reproducible *in vitro* model that could allow us to accurately study its role in PD pathogenesis. As such, it makes it even more difficult to screen for potential therapeutic compounds that could halt PD progression.

One obvious advantage of patient-derived iPSCs is that the iPSCs can be differentiated into disease-relevant DA neurons, and phenotypes observed in these *in vitro* neurons are well correlated with clinical observations (Torrent et al., 2015). Apart from being an endless source of midbrain DA neurons, using patient-derived cells (with their specific mutations) removes the need to rely on an artificial overexpression system that is not representative of PD pathology. Recent advances in the CRISPR/Cas9 technology has greatly availed genome-editing strategies to stem cell labs to create isogenic pairs of iPSCs. These typically take the form of “corrected iPSCs” where the disease-causing mutation is corrected to become wild-type, or “mutation-introduced iPSCs,” where wild-type iPSCs have their genomic DNA altered into a known disease-causing mutation (Soldner et al., 2011; Hockemeyer and Jaenisch, 2016; Bassett, 2017). The rationale for generating isogenic pairs of iPSCs is to minimize genetic variation that is inherent between different individuals and/or cell lines, and is crucial in disease modeling studies to identify disease-related molecular and cellular events.

Importantly, a-syn aggregation has been observed in DA neurons derived from PD iPSCs. By differentiating a-syn A53T iPSCs into midbrain DA neurons, Kouroupi et al. (2017) detected the presence of the pathological form of a-syn that is phosphorylated on serine 129 in the dendrites of PD neurons, reminiscent of Lewy neurites in PD patients (Kouroupi et al., 2017). Protein aggregates, as revealed by Thioflavin S staining, also showed high concentrations of a-syn and such inclusion bodies were observed in the cell bodies as well as along neurites. iPSCs derived from patients with PINK1 and Parkin mutations also differentiate into midbrain DA neurons that showed a time-dependent increased a-syn accumulation (Chung et al., 2016). It was also demonstrated that mutant PINK1 and Parkin DA neurons had significantly more insoluble a-syn protein, indicative of aggregated a-syn. These studies have proven that important cellular features of PD are recapitulated in iPSC-derived neurons, similar to what has been observed for other neurodegenerative diseases.

**Figure 1:**
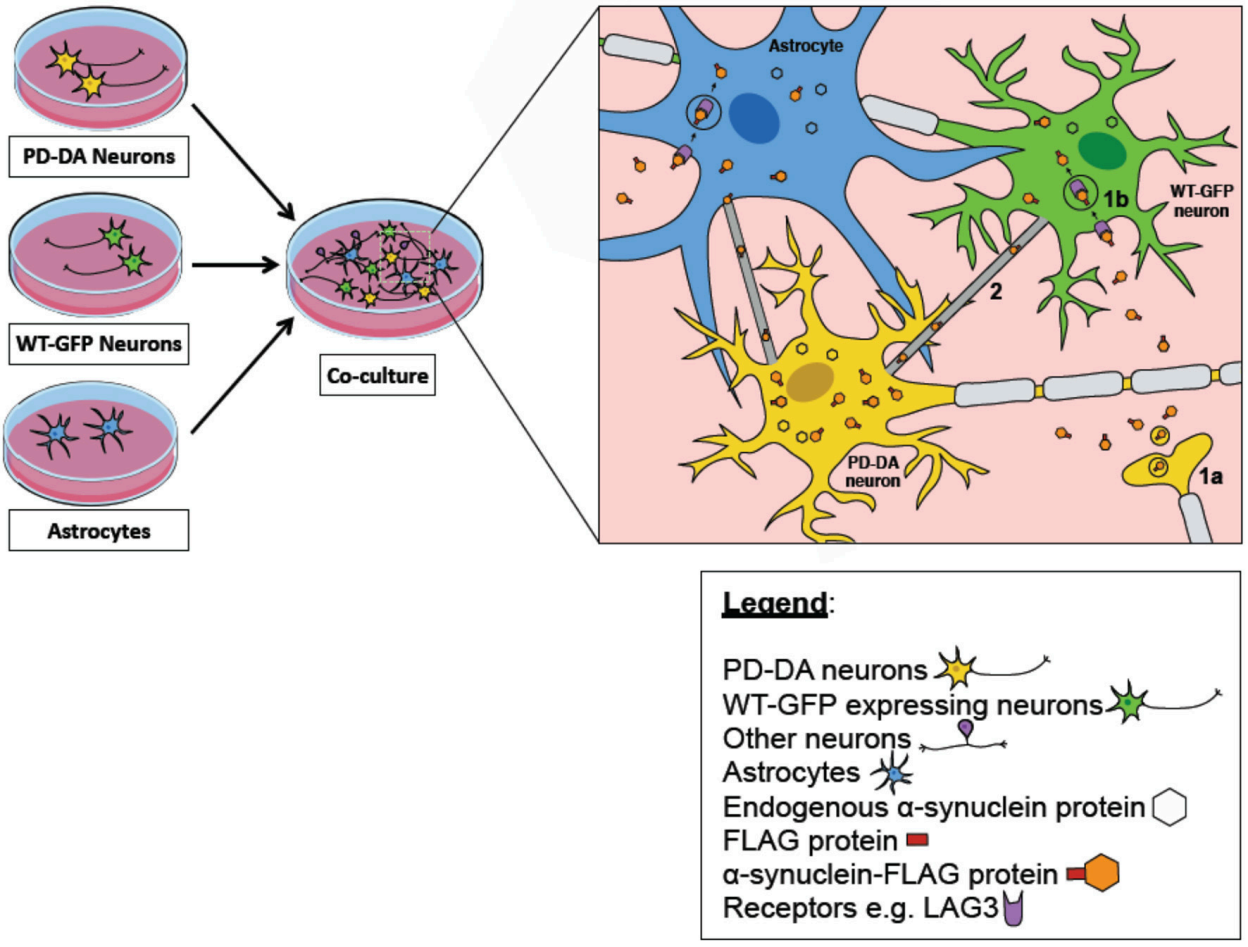
iPSC-derived midbrain cultures as an *in vitro* model of alpha synuclein transmission. A co-culture model of PD-DA neurons (cells in yellow), WT-GFP neurons (cells in green), and astrocytes (in blue) can be used to track the transfer of pathogenic alpha-synuclein (orange hexagon) between diseased and healthy neurons/astrocytes. PD-DA neurons are derived from the iPSCs of PD patients with their alpha synuclein tagged with a FLAG protein (red rectangle). WT-GFP neurons are derived from the iPSCs of healthy subjects and are constitutively expressing GFP as a reporter–the successful transmission of alpha-synuclein between diseased and healthy neurons can be defined as GFP-expressing cells co-expressing the FLAG signal. Several mechanisms have been postulated to be involved in the propagation of diseased alpha-synuclein to healthy neurons/astrocytes. One mechanism describes that pathogenic alpha synuclein secreted by PD-DA neurons (1a) could interact with various surface proteins on healthy neurons/astrocytes to induce uptake through receptor-mediated endocytosis (1b), for example LAG3 receptor. Furthermore, there are also specialized structures known as tunneling nanotubes (TNTs) between neuron-neuron and neuron-astrocytes that are involved in the spread of alpha synuclein (2).

Critically, what has not been elucidated in these iPSC studies is the transmission and propagation of endogenous a-syn aggregates. While important discoveries pertaining to mechanistic spread of a-syn have been made using exogenously-added PFFs, this approach over-simplifies the physiological conditions in which a-syn isoforms exist. Therefore, it remains to be determined if LAG3 or heparan sulfate proteoglycan reduction can protect neurons against a-syn propagation. It would also be important to establish that TNTs transport endogenously-formed a-syn aggregates from host to recipient cells in iNs or iPSC-derived cultures. We propose that these endogenous propagation studies can be performed by co-culturing PD iPSC-derived neural cultures with healthy neural cultures, either in 2D or as organoids (Figure 1). One possible method to track a-syn transfer from diseased to healthy cells, a-syn from PD patients has to be tagged with a small reporter protein such as Y-FAST (Plamont et al., 2016), while healthy cells should express a different reporter such as constitutive expression of CFP. Successful transmission events would then be defined as CFP-expressing cells co-stained with Y-FAST. It also remains to be discovered whether monomeric, oligomeric or fibrillar forms of a-syn are transmitted from host to recipient cells.

An advantage of organoid models vs. conventional 2D cultures is that the cytoarchitecture of cells in organoids closely resemble that of a brain—which may enhance neuronal maturation and/or function that promotes aggregate transmission. Since PD is an age-onset neurodegenerative disease, it is also likely that the maturation status of neurons is critical for a-syn transmission. Neurons grown in 3D cultures are also known to be more mature (Jo et al., 2016), and accumulate aggregates (Choi et al., 2014). The heterogeneity of neural cells in organoids is also an ideal system for studying selective neuronal vulnerability in PD. In particular, there are key questions left unanswered: Are specific neural types (astrocytes, DA neurons or other neuronal subtypes) more susceptible to a-syn transmission? What is the molecular signature of neurons with a-syn aggregates? Will attenuating key molecular events downstream of a-syn transmission protect neurons from cell death? Single-cell RNA-seq data of organoid-derived neural cells are expected to give us insights to some of these questions.

## Concluding remarks

Alpha synuclein accumulation, aggregation and transmission are key events in the pathology of PD, and strategies to prevent any of these events are thought to be able to slow down disease progression. Patient-derived iPSCs, coupled with the use of genome-editing tools, have become powerful tools in disease modeling, but its utility in modeling a-syn propagation has not been explored. In this review, we present a point-of-view that iNs and iPSC-derived neurons can be a physiologically relevant, all-in-one model that provides the opportunity to study a-syn accumulation, aggregation and transmission concurrently.

## Author contributions

All three authors wrote the manuscript. LYT designed the graphics. S-YN edited the manuscript.

### Conflict of interest statement

The authors declare that the research was conducted in the absence of any commercial or financial relationships that could be construed as a potential conflict of interest.

## References

[B1] Abounit S. Bousset L. Loria F. Zhu S. de Chaumont F. Pieri L.. (2016). Tunneling nanotubes spread fibrillar alpha-synuclein by intercellular trafficking of lysosomes. EMBO J. 35, 2120–2138. 10.15252/embj.201593411 27550960 PMC5048354

[B2] Addis R. C. Hsu F. C. Wright R. L. Dichter M. A. Coulter D. A. Gearhart J. D. (2011). Efficient conversion of astrocytes to functional midbrain dopaminergic neurons using a single polycistronic vector. PLoS ONE 6:e28719. 10.1371/journal.pone.0028719 22174877 PMC3235158

[B3] Alvarez-Erviti L. Seow Y. Schapira A. H. Gardiner C. Sargent I. L. Wood M. J.. (2011). Lysosomal dysfunction increases exosome-mediated alpha-synuclein release and transmission. Neurobiol Dis. 42, 360–367. 10.1016/j.nbd.2011.01.029 21303699 PMC3107939

[B4] Angot E. Steiner J. A. Lema Tomé C. M. Ekström P. Mattsson B. Björklund A.. (2012). Alpha-synuclein cell-to-cell transfer and seeding in grafted dopaminergic neurons *in vivo*. PLoS ONE 7:e39465. 10.1371/journal.pone.0039465 22737239 PMC3380846

[B5] Ardhanareeswaran K. Mariani J. Coppola G. Abyzov A. Vaccarino F. M. (2017). Human induced pluripotent stem cells for modelling neurodevelopmental disorders. Nat. Rev. Neurol. 13, 265–278. 10.1038/nrneurol.2017.45 28418023 PMC5782822

[B6] Ascherio A. Schwarzschild M. A. (2016). The epidemiology of Parkinson's disease: risk factors and prevention. Lancet Neurol. 15, 1257–1272. 10.1016/S1474-4422(16)30230-7 27751556

[B7] Ban H. Nishishita N. Fusaki N. Tabata T. Saeki K. Shikamura M.. (2011). Efficient generation of transgene-free human induced pluripotent stem cells (iPSCs) by temperature-sensitive Sendai virus vectors. Proc. Natl. Acad. Sci. U S A. 108, 14234–14239. 10.1073/pnas.1103509108 21821793 PMC3161531

[B8] Barker R. A. Studer L. Cattaneo E. Takahashi J. G-Force PD consortium (2015). G-Force PD: a global initiative in coordinating stem cell-based dopamine treatments for Parkinson's disease. NPJ Parkinsons Dis. 1:15017. 10.1038/npjparkd.2015.17 28725685 PMC5516551

[B9] Bassett A. R. (2017). Editing the genome of hiPSC with CRISPR/Cas9: disease models. Mamm. Genome. 28, 348–364. 10.1007/s00335-017-9684-9 28303292 PMC5569153

[B10] Blesa J. Przedborski S. (2014). Parkinson's disease: animal models and dopaminergic cell vulnerability. Front. Neuroanatomy 8:155. 10.3389/fnana.2014.00155 25565980 PMC4266040

[B11] Borghi R. Marchese R. Negro A. Marinelli L. Forloni G. Zaccheo D.. (2000). Full length alpha-synuclein is present in cerebrospinal fluid from Parkinson's disease and normal subjects. Neurosci. Lett. 287, 65–67. 10.1016/S0304-3940(00)01153-8 10841992

[B12] Braak H. Del Tredici K. Rüb U. de Vos R. A. Jansen Steur E. N. Braak E. (2003). Staging of brain pathology related to sporadic Parkinson's disease. Neurobiol. Aging. 24, 197–211. 10.1016/S0197-4580(02)00065-9 12498954

[B13] Braak H. Sastre M. Del Tredici K. (2007). Development of α-synuclein immunoreactive astrocytes in the forebrain parallels stages of intraneuronal pathology in sporadic Parkinson's disease. Acta Neuropathologica 114, 231–241. 10.1007/s00401-007-0244-3 17576580

[B14] Byers B. Cord B. Nguyen H. N. Schüle B. Fenno L. Lee P. C.. (2011). SNCA triplication Parkinson's patient's iPSC-derived DA neurons accumulate alpha-synuclein and are susceptible to oxidative stress. PLoS ONE 6:e26159. 10.1371/journal.pone.0026159 22110584 PMC3217921

[B15] Caiazzo M. Dell'Anno M. T. Dvoretskova E. Lazarevic D. Taverna S. Leo D.. (2011). Direct generation of functional dopaminergic neurons from mouse and human fibroblasts. Nature 476, 224–227. 10.1038/nature10284 21725324

[B16] Carey B. W. Markoulaki S. Hanna J. Saha K. Gao Q. Mitalipova M.. (2009). Reprogramming of murine and human somatic cells using a single polycistronic vector. Proc. Natl. Acad. Sci. U S A. 106, 157–162. 10.1073/pnas.0811426106 19109433 PMC2629226

[B17] Chen G. Gulbranson D. R. Hou Z. Bolin J. M. Ruotti V. Probasco M. D.. (2011). Chemically defined conditions for human iPSC derivation and culture. Nat. Methods 8, 424–429. 10.1038/nmeth.1593 21478862 PMC3084903

[B18] Choi S. H. Kim Y. H. Hebisch M. Sliwinski C. Lee S. D'Avanzo C.. (2014). A three-dimensional human neural cell culture model of Alzheimer's disease. Nature 515, 274–278. 10.1038/nature13800 25307057 PMC4366007

[B19] Chung S. Y. Kishinevsky S. Mazzulli J. R. Graziotto J. Mrejeru A. Mosharov E. V.. (2016). Parkin and PINK1 patient iPSC-derived midbrain dopamine neurons exhibit mitochondrial dysfunction and alpha-synuclein accumulation. Stem Cell Rep. 7, 664–677. 10.1016/j.stemcr.2016.08.012 27641647 PMC5063469

[B20] Cooper O. Hargus G. Deleidi M. Blak A. Osborn T. Marlow E.. (2010). Differentiation of human ES and Parkinson's disease iPS cells into ventral midbrain dopaminergic neurons requires a high activity form of SHH, FGF8a and specific regionalization by retinoic acid. Mol. Cell. Neurosci. 45, 258–266. 10.1016/j.mcn.2010.06.017 20603216 PMC2945816

[B21] Cooper O. Seo H. Andrabi S. Guardia-Laguarta C. Graziotto J. Sundberg M.. (2012). Pharmacological rescue of mitochondrial deficits in iPSC-derived neural cells from patients with familial Parkinson's disease. Sci. Transl. Med. 4:141ra90. 10.1126/scitranslmed.3003985 22764206 PMC3462009

[B22] Cuervo A. M. Stefanis L. Fredenburg R. Lansbury P. T. Sulzer D. (2004). Impaired degradation of mutant alpha-synuclein by chaperone-mediated autophagy. Science 305, 1292–1295. 10.1126/science.1101738 15333840

[B23] Danzer K. M. Kranich L. R. Ruf W. P. Cagsal-Getkin O. Winslow A. R. Zhu L.. (2012). Exosomal cell-to-cell transmission of alpha synuclein oligomers. Mol. Neurodegener. 7:42. 10.1186/1750-1326-7-42 22920859 PMC3483256

[B24] Desplats P. Lee H. J. Bae E. J. Patrick C. Rockenstein E. Crews L.. (2009). Inclusion formation and neuronal cell death through neuron-to-neuron transmission of alpha-synuclein. Proc. Natl. Acad. Sci. U S A. 106, 13010–13015. 10.1073/pnas.0903691106 19651612 PMC2722313

[B25] Devi L. Raghavendran V. Prabhu B. M. Avadhani N. G. Anandatheerthavarada H. K. (2008). Mitochondrial import and accumulation of alpha-synuclein impair complex I in human dopaminergic neuronal cultures and Parkinson disease brain. J. Biol. Chem. 283, 9089–9100. 10.1074/jbc.M710012200 18245082 PMC2431021

[B26] Devine M. J. Ryten M. Vodicka P. Thomson A. J. Burdon T. Houlden H.. (2011). Parkinson's disease induced pluripotent stem cells with triplication of the alpha-synuclein locus. Nat. Commun. 2:440. 10.1038/ncomms1453 21863007 PMC3265381

[B27] Di Giovannantonio L. G. Di Salvio M. Acampora D. Prakash N. Wurst W. Simeone A. (2013). Otx2 selectively controls the neurogenesis of specific neuronal subtypes of the ventral tegmental area and compensates En1-dependent neuronal loss and MPTP vulnerability. Dev. Biol. 373, 176–183. 10.1016/j.ydbio.2012.10.022 23117062

[B28] Doi D. Samata B. Katsukawa M. Kikuchi T. Morizane A. Ono Y.. (2014). Isolation of human induced pluripotent stem cell-derived dopaminergic progenitors by cell sorting for successful transplantation. Stem Cell Rep. 2, 337–350. 10.1016/j.stemcr.2014.01.013 24672756 PMC3964289

[B29] El-Agnaf O. M. Salem S. A. Paleologou K. E. Curran M. D. Gibson M. J. Court J. A.. (2006). Detection of oligomeric forms of alpha-synuclein protein in human plasma as a potential biomarker for Parkinson's disease. FASEB J. 20, 419–425. 10.1096/fj.03-1449com 16507759

[B30] Emmanouilidou E. Melachroinou K. Roumeliotis T. Garbis S. D. Ntzouni M. Margaritis L. H.. (2010). Cell-produced alpha-synuclein is secreted in a calcium-dependent manner by exosomes and impacts neuronal survival. J. Neurosci. 30, 6838–6851. 10.1523/JNEUROSCI.5699-09.2010 20484626 PMC3842464

[B31] Evans M. (2011). Discovering pluripotency: 30 years of mouse embryonic stem cells. Nat. Rev. Mol. Cell Biol. 12, 680–686. 10.1038/nrm3190 21941277

[B32] Fasano C. A. Chambers S. M. Lee G. Tomishima M. J. Studer L. (2010). Efficient derivation of functional floor plate tissue from human embryonic stem cells. Cell Stem Cell 6, 336–347. 10.1016/j.stem.2010.03.001 20362538 PMC4336800

[B33] Fellner L. Irschick R. Schanda K. Reindl M. Klimaschewski L. Poewe W.. (2013). Toll-like receptor 4 is required for α-synuclein dependent activation of microglia and astroglia. Glia 61, 349–360. 10.1002/glia.22437 23108585 PMC3568908

[B34] Fernandes H. J. Hartfield E. M. Christian H. C. Emmanoulidou E. Zheng Y. Booth H.. (2016). ER stress and autophagic perturbations lead to elevated extracellular alpha-synuclein in GBA-N370S parkinson's iPSC-derived dopamine neurons. Stem Cell Rep. 6, 342–356. 10.1016/j.stemcr.2016.01.013 26905200 PMC4788783

[B35] Flierl A. Oliveira L. M. Falomir-Lockhart L. J. Mak S. K. Hesley J. Soldner F.. (2014). Higher vulnerability and stress sensitivity of neuronal precursor cells carrying an alpha-synuclein gene triplication. PLoS ONE 9:e112413. 10.1371/journal.pone.0112413 25390032 PMC4229205

[B36] Fusaki N. Ban H. Nishiyama A. Saeki K. Hasegawa M. (2009). Efficient induction of transgene-free human pluripotent stem cells using a vector based on Sendai virus, an RNA virus that does not integrate into the host genome. Proc. Jpn Acad. Ser. B Phys. Biol. Sci. 85, 348–362. 10.2183/pjab.85.348 PMC3621571 19838014

[B37] Hansen C. Angot E. Bergström A. L. Steiner J. A. Pieri L. Paul G.. (2011). alpha-Synuclein propagates from mouse brain to grafted dopaminergic neurons and seeds aggregation in cultured human cells. J. Clin. Invest. 121, 715–725. 10.1172/JCI43366 21245577 PMC3026723

[B38] Heinrich C. Bergami M. Gascón S. Lepier A. Viganò F. Dimou L.. (2014). Sox2-mediated conversion of NG2 glia into induced neurons in the injured adult cerebral cortex. Stem Cell Rep. 3, 1000–1014. 10.1016/j.stemcr.2014.10.007 25458895 PMC4264057

[B39] Hockemeyer D. Jaenisch R. (2016). Induced pluripotent stem cells meet genome editing. Cell Stem Cell 18, 573–586. 10.1016/j.stem.2016.04.013 27152442 PMC4871596

[B40] Holmes B. B. DeVos S. L. Kfoury N. Li M. Jacks R. Yanamandra K.. (2013). Heparan sulfate proteoglycans mediate internalization and propagation of specific proteopathic seeds. Proc. Natl. Acad. Sci. U.S.A. 110, E3138–E3147. 10.1073/pnas.1301440110 23898162 PMC3746848

[B41] Hou P. Li Y. Zhang X. Liu C. Guan J. Li H.. (2013). Pluripotent stem cells induced from mouse somatic cells by small-molecule compounds. Science 341, 651–654. 10.1126/science.1239278 23868920

[B42] Huh C. J. Zhang B. Victor M. B. Dahiya S. Batista L. F. Horvath S.. (2016). Maintenance of age in human neurons generated by microRNA-based neuronal conversion of fibroblasts. Elife 5:e18648. 10.7554/eLife.18648 27644593 PMC5067114

[B43] Imaizumi Y. Okada Y. Akamatsu W. Koike M. Kuzumaki N. Hayakawa H.. (2012). Mitochondrial dysfunction associated with increased oxidative stress and alpha-synuclein accumulation in PARK2 iPSC-derived neurons and postmortem brain tissue. Mol. Brain. 5:35. 10.1186/1756-6606-5-35 23039195 PMC3546866

[B44] Jang Y. Jung J. H. (2017). Direct conversion from skin fibroblasts to functional dopaminergic neurons for biomedical application. Biomed. Dermatol. 1:4 10.1186/s41702-017-0004-5

[B45] Jiang H. Xu Z. Zhong P. Ren Y. Liang G. Schilling H. A.. (2015). Cell cycle and p53 gate the direct conversion of human fibroblasts to dopaminergic neurons. Nat. Commun. 6:10100. 10.1038/ncomms10100 26639555 PMC4672381

[B46] Jo J. Xiao Y. Sun A. X. Cukuroglu E. Tran H. D. Göke J.. (2016). Midbrain-like organoids from human pluripotent stem cells contain functional dopaminergic and neuromelanin-producing neurons. Cell Stem Cell 19, 248–257. 10.1016/j.stem.2016.07.005 27476966 PMC5510242

[B47] Kaji K. Norrby K. Paca A. Mileikovsky M. Mohseni P. Woltjen K. (2009). Virus-free induction of pluripotency and subsequent excision of reprogramming factors. Nature 458, 771–775. 10.1038/nature07864 19252477 PMC2667910

[B48] Kamp F. Exner N. Lutz A. K. Wender N. Hegermann J. Brunner B.. (2010). Inhibition of mitochondrial fusion by alpha-synuclein is rescued by PINK1, Parkin and DJ-1. EMBO J. 29, 3571–3589. 10.1038/emboj.2010.223 20842103 PMC2964170

[B49] Kanaan N. M. Manfredsson F. P. (2012). Loss of functional alpha-synuclein: a toxic event in Parkinson's disease? J. Parkinsons. Dis. 2, 249–267. 10.3233/JPD-012138 23938255 PMC4736738

[B50] Karpinar D. P. Balija M. B. Kugler S. Opazo F. Rezaei-Ghaleh N. Wender N.. (2009). Pre-fibrillar alpha-synuclein variants with impaired beta-structure increase neurotoxicity in Parkinson's disease models. EMBO J. 28, 3256–3268. 10.1038/emboj.2009.257 19745811 PMC2771093

[B51] Kawasaki H. Mizuseki K. Nishikawa S. Kaneko S. Kuwana Y. Nakanishi S.. (2000). Induction of midbrain dopaminergic neurons from ES cells by stromal cell-derived inducing activity. Neuron 28, 31–40. 10.1016/S0896-6273(00)00083-0 11086981

[B52] Kim D. Kim C. H. Moon J. I. Chung Y. G. Chang M. Y. Han B. S.. (2009). Generation of human induced pluripotent stem cells by direct delivery of reprogramming proteins. Cell Stem Cell 4, 472–476. 10.1016/j.stem.2009.05.005 19481515 PMC2705327

[B53] Kim J. Su S. C. Wang H. Cheng A. W. Cassady J. P. Lodato M. A.. (2011). Functional integration of dopaminergic neurons directly converted from mouse fibroblasts. Cell Stem Cell 9, 413–419. 10.1016/j.stem.2011.09.011 22019014 PMC3210333

[B54] Kirkeby A. Grealish S. Wolf D. A. Nelander J. Wood J. Lundblad M.. (2012). Generation of regionally specified neural progenitors and functional neurons from human embryonic stem cells under defined conditions. Cell Rep. 1, 703–714. 10.1016/j.celrep.2012.04.009 22813745

[B55] Kirkeby A. Parmar M. (2012). Building authentic midbrain dopaminergic neurons from stem cells - lessons from development. Transl. Neurosci. 3, 314–319. 10.2478/s13380-012-0041-x

[B56] Kisos H. Pukaß K. Ben-Hur T. Richter-Landsberg C. Sharon R. (2012). Increased neuronal α-synuclein pathology associates with its accumulation in oligodendrocytes in mice modeling α-synucleinopathies. PLoS ONE 7:e46817. 10.1371/journal.pone.0046817 23077527 PMC3471961

[B57] Klein C. Westenberger A. (2012). Genetics of Parkinson's disease. Cold Spring Harb. Perspect. Med. 2:a008888. 10.1101/cshperspect.a008888 22315721 PMC3253033

[B58] Konno M. Hasegawa T. Baba T. Miura E. Sugeno N. Kikuchi A.. (2012). Suppression of dynamin GTPase decreases α-synuclein uptake by neuronal and oligodendroglial cells: a potent therapeutic target for synucleinopathy. Mol. Neurodegener. 7:38. 10.1186/1750-1326-7-38 22892036 PMC3479026

[B59] Kordower J. H. Chu Y. Hauser R. A. Freeman T. B. Olanow C. W. (2008). Lewy body-like pathology in long-term embryonic nigral transplants in Parkinson's disease. Nat. Med. 14, 504–506. 10.1038/nm1747 18391962

[B60] Kordower J. H. Dodiya H. B. Kordower A. M. Terpstra B. Paumier K. Madhavan L.. (2011). Transfer of host-derived alpha synuclein to grafted dopaminergic neurons in rat. Neurobiol. Dis. 43, 552–557. 10.1016/j.nbd.2011.05.001 21600984 PMC3430516

[B61] Kouroupi G. Taoufik E. Vlachos I. S. Tsioras K. Antoniou N. Papastefanaki F.. (2017). Defective synaptic connectivity and axonal neuropathology in a human iPSC-based model of familial Parkinson's disease. Proc. Natl. Acad. Sci. USA. 114, E3679–E88. 10.1073/pnas.1617259114 28416701 PMC5422768

[B62] Kriks S. Shim J. W. Piao J. Ganat Y. M. Wakeman D. R. Xie Z.. (2011). Dopamine neurons derived from human ES cells efficiently engraft in animal models of Parkinson's disease. Nature 480, 547–551. 10.1038/nature10648 22056989 PMC3245796

[B63] Lancaster M. A. Knoblich J. A. (2014). Generation of cerebral organoids from human pluripotent stem cells. Nat. Protoc. 9, 2329–2340. 10.1038/nprot.2014.158 25188634 PMC4160653

[B64] Lancaster M. A. Renner M. Martin C. A. Wenzel D. Bicknell L. S. Hurles M. E.. (2013). Cerebral organoids model human brain development and microcephaly. Nature 501, 373–379. 10.1038/nature12517 23995685 PMC3817409

[B65] Lashuel H. A. Overk C. R. Oueslati A. Masliah E. (2012). The many faces of α-synuclein: from structure and toxicity to therapeutic target. Nat. Rev. Neurosci. 14:38–48. 10.1038/nrn3406 23254192 PMC4295774

[B66] Lee H. J. Cho E. D. Lee K. W. Kim J. H. Cho S. G. Lee S. J. (2013). Autophagic failure promotes the exocytosis and intercellular transfer of alpha-synuclein. Exp. Mol. Med. 45:e22. 10.1038/emm.2013.45 23661100 PMC3674407

[B67] Lee H. J. Suk J. E. Patrick C. Bae E. J. Cho J. H. Rho S.. (2010). Direct transfer of alpha-synuclein from neuron to astroglia causes inflammatory responses in synucleinopathies. J. Biol. Chem. 285, 9262–9272. 10.1074/jbc.M109.081125 20071342 PMC2838344

[B68] Lee P. H. Lee G. Park H. J. Bang O. Y. Joo I. S. Huh K. (2006). The plasma alpha-synuclein levels in patients with Parkinson's disease and multiple system atrophy. J. Neural. Transm. 113, 1435–1439. 10.1007/s00702-005-0427-9 16465458

[B69] Li J. Y. Englund E. Holton J. L. Soulet D. Hagell P. Lees A. J.. (2008). Lewy bodies in grafted neurons in subjects with Parkinson's disease suggest host-to-graft disease propagation. Nat. Med. 14, 501–503. 10.1038/nm1746 18391963

[B70] Lindström V. Gustafsson G. Sanders L. H. Howlett E. H. Sigvardson J. Kasrayan A.. (2017). Extensive uptake of α-synuclein oligomers in astrocytes results in sustained intracellular deposits and mitochondrial damage. Mol. Cell. Neurosci. 82, 143–156. 10.1016/j.mcn.2017.04.009 28450268

[B71] Liu X. Li F. Stubblefield E. A. Blanchard B. Richards T. L. Larson G. A.. (2012). Direct reprogramming of human fibroblasts into dopaminergic neuron-like cells. Cell Res. 22, 321–332. 10.1038/cr.2011.181 22105488 PMC3271588

[B72] Luk K. C. Kehm V. Carroll J. Zhang B. O'Brien P. Trojanowski J. Q.. (2012a). Pathological alpha-synuclein transmission initiates Parkinson-like neurodegeneration in nontransgenic mice. Science 338, 949–953. 10.1126/science.1227157 23161999 PMC3552321

[B73] Luk K. C. Kehm V. M. Zhang B. O'Brien P. Trojanowski J. Q. Lee V. M. (2012b). Intracerebral inoculation of pathological alpha-synuclein initiates a rapidly progressive neurodegenerative alpha-synucleinopathy in mice. J. Exp. Med. 209, 975–986. 10.1084/jem.20112457 22508839 PMC3348112

[B74] Malik N. Rao M. S. (2013). A review of the methods for human iPSC derivation. Methods Mol. Biol. 997:23–33. 10.1007/978-1-62703-348-0_3 23546745 PMC4176696

[B75] Mao X. Ou M. T. Karuppagounder S. S. Kam T. I. Yin X. Xiong Y.. (2016). Pathological alpha-synuclein transmission initiated by binding lymphocyte-activation gene 3. Science 353:aah3374. 10.1126/science.aah3374 27708076 PMC5510615

[B76] Marton R. M. Paşca S. P. (2016). Neural differentiation in the third dimension: generating a human midbrain. Cell Stem Cell 19, 145–146. 10.1016/j.stem.2016.07.017 27494668

[B77] Masuda-Suzukake M. Nonaka T. Hosokawa M. Kubo M. Shimozawa A. Akiyama H.. (2014). Pathological alpha-synuclein propagates through neural networks. Acta Neuropathologica Commun. 2:88. 10.1186/s40478-014-0088-8 25095794 PMC4147188

[B78] Mertens J. Paquola A. C. Ku M. Hatch E. Böhnke L. Ladjevardi S.. (2015). Directly reprogrammed human neurons retain aging-associated transcriptomic signatures and reveal age-related nucleocytoplasmic defects. Cell Stem Cell 17, 705–718. 10.1016/j.stem.2015.09.001 26456686 PMC5929130

[B79] Miller D. Johnson J. Solano S. Hollingsworth Z. Standaert D. Young A. (2005). Absence of α-synuclein mRNA expression in normal and multiple system atrophy oligodendroglia. J. Neural Transm. 112, 1613–1624. 10.1007/s00702-005-0378-1 16284907

[B80] Miyoshi N. Ishii H. Nagano H. Haraguchi N. Dewi D. L Kano Y.. (2011). Reprogramming of mouse and human cells to pluripotency using mature microRNAs. Cell Stem Cell 8, 633–638. 10.1016/j.stem.2011.05.001 21620789

[B81] Monzel A. S. Smits L. M. Hemmer K. Hachi S. Moreno E. L. van Wuellen T.. (2017). Derivation of human midbrain-specific organoids from neuroepithelial stem cells. Stem Cell Rep. 8, 1144–1154. 10.1016/j.stemcr.2017.03.010 28416282 PMC5425618

[B82] Mougenot A. L. Nicot S. Bencsik A. Morignat E. Verchère J. Lakhdar L.. (2012). Prion-like acceleration of a synucleinopathy in a transgenic mouse model. Neurobiol. Aging 33, 2225–2228. 10.1016/j.neurobiolaging.2011.06.022 21813214

[B83] Muguruma K. Nishiyama A. Kawakami H. Hashimoto K. Sasai Y. (2015). Self-organization of polarized cerebellar tissue in 3D culture of human pluripotent stem cells. Cell Rep. 10, 537–550. 10.1016/j.celrep.2014.12.051 25640179

[B84] Nakamura K. Nemani V. M. Azarbal F. Skibinski G. Levy J. M. Egami K.. (2011). Direct membrane association drives mitochondrial fission by the Parkinson disease-associated protein alpha-synuclein. J. Biol. Chem. 286, 20710–20726. 10.1074/jbc.M110.213538 21489994 PMC3121472

[B85] Paik E. J. O'Neil A. L. Ng S. Y. Sun C. Rubin L. L. (2018). Using intracellular markers to identify a novel set of surface markers for live cell purification from a heterogeneous hIPSC culture. Sci. Rep. 8, 804. 10.1038/s41598-018-19291-4 29339826 PMC5770419

[B86] Pang Z. P. Yang N. Vierbuchen T. Ostermeier A. Fuentes D. R. Yang T. Q.. (2011). Induction of human neuronal cells by defined transcription factors. Nature 476:220–223. 10.1038/nature10202 21617644 PMC3159048

[B87] Panman L. Papathanou M. Laguna A. Oosterveen T. Volakakis N. Acampora D.. (2014). Sox6 and Otx2 control the specification of substantia nigra and ventral tegmental area dopamine neurons. Cell Rep. 8, 1018–1025. 10.1016/j.celrep.2014.07.016 25127144

[B88] Pan-Montojo F. Anichtchik O. Dening Y. Knels L. Pursche S. Jung R.. (2010). Progression of Parkinson's disease pathology is reproduced by intragastric administration of rotenone in mice. PLoS ONE 5:e8762. 10.1371/journal.pone.0008762 20098733 PMC2808242

[B89] Pan-Montojo F. Schwarz M. Winkler C. Arnhold M. O'Sullivan G. A. Pal A.. (2012). Environmental toxins trigger PD-like progression via increased alpha-synuclein release from enteric neurons in mice. Sci. Rep. 2:898. 10.1038/srep00898 23205266 PMC3510466

[B90] Peelaerts W. Bousset L. Van der Perren A. Moskalyuk A. Pulizzi R. Giugliano M.. (2015). alpha-Synuclein strains cause distinct synucleinopathies after local and systemic administration. Nature 522, 340–344. 10.1038/nature14547 26061766

[B91] Perrier A. L. Tabar V. Barberi T. Rubio M. E. Bruses J. Topf N.. (2004). Derivation of midbrain dopamine neurons from human embryonic stem cells. Proc. Natl. Acad. Sci. U.S.A. 101, 12543–12548. 10.1073/pnas.0404700101 15310843 PMC515094

[B92] Pfisterer U. Kirkeby A. Torper O. Wood J. Nelander J. Dufour A.. (2011). Direct conversion of human fibroblasts to dopaminergic neurons. Proc. Natl. Acad. Sci. USA. 108, 10343–10348. 10.1073/pnas.1105135108 21646515 PMC3121829

[B93] Plamont M. A. Billon-Denis E. Maurin S. Gauron C. Pimenta F. M. Specht C. G.. (2016). Small fluorescence-activating and absorption-shifting tag for tunable protein imaging *in vivo*. Proc. Natl. Acad. Sci. USA. 113, 497–502. 10.1073/pnas.1513094113 26711992 PMC4725535

[B94] Poulin J. F. Zou J. Drouin-Ouellet J. Kim K-Y A. Cicchetti F. Awatramani R. B. (2014). Defining midbrain dopaminergic neuron diversity by single-cell gene expression profiling. Cell Rep. 9, 930–943. 10.1016/j.celrep.2014.10.008 25437550 PMC4251558

[B95] Qian X. Nguyen H. N. Song M. M. Hadiono C. Ogden S. C. Hammack C.. (2016). Brain region-specific organoids using mini-bioreactors for modeling ZIKV exposure. Cell 165, 1238–1254. 10.1016/j.cell.2016.04.032 27118425 PMC4900885

[B96] Rannikko E. H. Weber S. S. Kahle P. J. (2015). Exogenous α-synuclein induces toll-like receptor 4 dependent inflammatory responses in astrocytes. BMC Neurosci. 16:57. 10.1186/s12868-015-0192-0 26346361 PMC4562100

[B97] Recasens A. Dehay B. Bové J. Carballo-Carbajal I. Dovero S. Pérez-Villalba A.. (2014). Lewy body extracts from Parkinson disease brains trigger alpha-synuclein pathology and neurodegeneration in mice and monkeys. Ann. Neurol. 75, 351–362. 10.1002/ana.24066 24243558

[B98] Rey N. L. Petit G. H. Bousset L. Melki R. Brundin P. (2013). Transfer of human alpha-synuclein from the olfactory bulb to interconnected brain regions in mice. Acta Neuropathol. 126, 555–573. 10.1007/s00401-013-1160-3 23925565 PMC3789892

[B99] Reyes J. F. Rey N. L. Bousset L. Melki R. Brundin P. Angot E. (2014). Alpha-synuclein transfers from neurons to oligodendrocytes. Glia 62, 387–398. 10.1002/glia.22611 24382629

[B100] Rostami J. Holmqvist S. Lindström V. Sigvardson J. Westermark G. T. Ingelsson M.. (2017). Human astrocytes transfer aggregated alpha-synuclein via tunneling nanotubes. J. Neurosci. 37, 11835–11853. 10.1523/JNEUROSCI.0983-17.2017 29089438 PMC5719970

[B101] Ryan S. D. Dolatabadi N. Chan S. F. Zhang X. Akhtar M. W. Parker J.. (2013). Isogenic human iPSC Parkinson's model shows nitrosative stress-induced dysfunction in MEF2-PGC1alpha transcription. Cell 155, 1351–1364. 10.1016/j.cell.2013.11.009 24290359 PMC4028128

[B102] Sacino A. N. Brooks M. McGarvey N. H. McKinney A. B. Thomas M. A. Levites Y.. (2013). Induction of CNS alpha-synuclein pathology by fibrillar and non-amyloidogenic recombinant alpha-synuclein. Acta Neuropathol. Commun. 1:38. 10.1186/2051-5960-1-38 24252149 PMC3893388

[B103] Seki T. Fukuda K. (2015). Methods of induced pluripotent stem cells for clinical application. World J. Stem Cells 7, 116–125. 10.4252/wjsc.v7.i1.116 25621111 PMC4300922

[B104] Shaltouki A. Sivapatham R. Pei Y. Gerencser A. A. Momcilović O. Rao M. S.. (2015). Mitochondrial alterations by PARKIN in dopaminergic neurons using PARK2 patient-specific and PARK2 knockout isogenic iPSC lines. Stem Cell Rep. 4, 847–859. 10.1016/j.stemcr.2015.02.019 25843045 PMC4437475

[B105] Sharon R. Goldberg M. S. Bar-Josef I. Betensky R. A. Shen J. Selkoe D. J. (2001). alpha-Synuclein occurs in lipid-rich high molecular weight complexes, binds fatty acids, and shows homology to the fatty acid-binding proteins. Proc. Natl. Acad. Sci. USA. 98, 9110–9115. 10.1073/pnas.171300598 11481478 PMC55381

[B106] Shimozawa A. Ono M. Takahara D. Tarutani A. Imura S. Masuda-Suzukake M.. (2017). Propagation of pathological alpha-synuclein in marmoset brain. Acta Neuropathol. Commun. 5:12. 10.1186/s40478-017-0413-0 28148299 PMC5289012

[B107] Snyder H. Mensah K. Theisler C. Lee J. Matouschek A. Wolozin B. (2003). Aggregated and monomeric alpha-synuclein bind to the S6' proteasomal protein and inhibit proteasomal function. J. Biol. Chem. 278, 11753–11759. 10.1074/jbc.M208641200 12551928

[B108] Soldner F. Laganière J. Cheng A. W. Hockemeyer D. Gao Q. Alagappan R.. (2011). Generation of isogenic pluripotent stem cells differing exclusively at two early onset Parkinson point mutations. Cell 146, 318–331. 10.1016/j.cell.2011.06.019 21757228 PMC3155290

[B109] Somers A. Jean J. C. Sommer C. A. Omari A. Ford C. C. Mills J. A. (2010). Generation of transgene-free lung disease-specific human iPS cells using a single excisable lentiviral stem cell cassette. Stem Cells 28, 1728–1740. 10.1002/stem.495 20715179 PMC3422663

[B110] Sommer C. A. Stadtfeld M. Murphy G. J. Hochedlinger K. Kotton D. N. Mostoslavsky G. (2009). iPS cell generation using a single lentiviral stem cell cassette. Stem Cells 27, 543–549. 10.1634/stemcells.2008-1075 19096035 PMC4848035

[B111] Stefanis L. (2012). alpha-synuclein in Parkinson's disease. Cold Spring Harb. Perspect. Med. 2:a009399. 10.1101/cshperspect.a009399 22355802 PMC3281589

[B112] Stefanis L. Larsen K. E. Rideout H. J. Sulzer D. Greene L. A. (2001). Expression of A53T mutant but not wild-type alpha-synuclein in PC12 cells induces alterations of the ubiquitin-dependent degradation system, loss of dopamine release, and autophagic cell death. J. Neurosci. 21, 9549–9560. 10.1523/JNEUROSCI.21-24-09549.2001 11739566 PMC6763041

[B113] Stefanova N. Fellner L. Reindl M. Masliah E. Poewe W. Wenning G. K. (2011). Toll-like receptor 4 promotes α-synuclein clearance and survival of nigral dopaminergic neurons. Am. J. Pathol. 179, 954–963. 10.1016/j.ajpath.2011.04.013 21801874 PMC3157205

[B114] Stuendl A. Kunadt M. Kruse N. Bartels C. Moebius W. Danzer K. M.. (2016). Induction of alpha-synuclein aggregate formation by CSF exosomes from patients with Parkinson's disease and dementia with Lewy bodies. Brain 139(Pt 2), 481–494. 10.1093/brain/awv346 26647156 PMC4805087

[B115] Subramanyam D. Lamouille S. Judson R. L. Liu J. Y. Bucay N. Derynck R.. (2011). Multiple targets of miR-302 and miR-372 promote reprogramming of human fibroblasts to induced pluripotent stem cells. Nat. Biotechnol. 29, 443–448. 10.1038/nbt.1862 21490602 PMC3685579

[B116] Takahashi K. Yamanaka S. (2006). Induction of pluripotent stem cells from mouse embryonic and adult fibroblast cultures by defined factors. Cell 126, 663–676. 10.1016/j.cell.2006.07.024 16904174

[B117] Tanaka Y. Engelender S. Igarashi S. Rao R. K. Wanner T. Tanzi R. E.. (2001). Inducible expression of mutant alpha-synuclein decreases proteasome activity and increases sensitivity to mitochondria-dependent apoptosis. Hum. Mol. Genet. 10, 919–926. 10.1093/hmg/10.9.919 11309365

[B118] Thakur P. Breger L. S. Lundblad M. Wan O. W. Mattsson B. Luk K. C.. (2017). Modeling Parkinson's disease pathology by combination of fibril seeds and alpha-synuclein overexpression in the rat brain. Proc. Natl. Acad. Sci. USA. 114, E8284–E93. 10.1073/pnas.1710442114 28900002 PMC5625925

[B119] Tian C. Li Y. Huang Y. Wang Y. Chen D. Liu J.. (2015). Selective generation of dopaminergic precursors from mouse fibroblasts by direct lineage conversion. Sci. Rep. 5:12622. 10.1038/srep12622 26224135 PMC4519786

[B120] Tokuda T. Salem S. A. Allsop D. Mizuno T. Nakagawa M. Qureshi M. M.. (2006). Decreased alpha-synuclein in cerebrospinal fluid of aged individuals and subjects with Parkinson's disease. Biochem. Biophys. Res. Commun. 349, 162–166. 10.1016/j.bbrc.2006.08.024 16930553

[B121] Torrent R. De Angelis Rigotti F. Dell'Era P. Memo M. Raya A. Consiglio A. (2015). Using iPS cells toward the understanding of Parkinson's disease. J. Clin. Med. 4, 548–566. 10.3390/jcm4040548 26239346 PMC4470155

[B122] Ubhi K. Low P. Masliah E. (2011). Multiple system atrophy: a clinical and neuropathological perspective. Trends Neurosci. 34, 581–590. 10.1016/j.tins.2011.08.003 21962754 PMC3200496

[B123] Vierbuchen T. Ostermeier A. Pang Z. P. Kokubu Y. Südhof T. C. Wernig M. (2010). Direct conversion of fibroblasts to functional neurons by defined factors. Nature 463, 1035–1041. 10.1038/nature08797 20107439 PMC2829121

[B124] Warren L. Manos P. D. Ahfeldt T. Loh Y.-H. Li H. Lau F.. (2010). Highly efficient reprogramming to pluripotency and directed differentiation of human cells using synthetic modified mRNA. Cell Stem Cell 7, 618–630. 10.1016/j.stem.2010.08.012 20888316 PMC3656821

[B125] Winner B. Jappelli R. Maji S. K. Desplats P. A. Boyer L. Aigner S.. (2011). *In vivo* demonstration that alpha-synuclein oligomers are toxic. Proc. Natl. Acad. Sci. U.S.A. 108, 4194–4199. 10.1073/pnas.1100976108 21325059 PMC3053976

[B126] Woltjen K. Michael I. P. Mohseni P. Desai R. Mileikovsky M. Hämäläinen R.. (2009). piggyBac transposition reprograms fibroblasts to induced pluripotent stem cells. Nature 458, 766–770. 10.1038/nature07863 19252478 PMC3758996

[B127] Xilouri M. Vogiatzi T. Vekrellis K. Park D. Stefanis L. (2009). Abberant alpha-synuclein confers toxicity to neurons in part through inhibition of chaperone-mediated autophagy. PLoS ONE 4:e5515. 10.1371/journal.pone.0005515 19436756 PMC2677735

[B128] Xu Z. Chu X. Jiang H. Schilling H. Chen S. Feng J. (2017). Induced dopaminergic neurons: a new promise for Parkinson's disease. Redox Biol. 11:606–612. 10.1016/j.redox.2017.01.009 28110217 PMC5256671

[B129] Yu J. Hu K. Smuga-Otto K. Tian S. Stewart R. Slukvin I. I.. (2009). Human induced pluripotent stem cells free of vector and transgene sequences. Science 324, 797–801. 10.1126/science.1172482 19325077 PMC2758053

[B130] Yu W.-W. Cao S.-N. Zang C.-X. Wang L. Yang H.-Y. Bao X.-Q.. (2018). Heat shock protein 70 suppresses neuroinflammation induced by α-synuclein in astrocytes. Mol. Cell. Neurosci. 86:58–64. 10.1016/j.mcn.2017.11.013 29183796

[B131] Yusa K. Rad R. Takeda J. Bradley A. (2009). Generation of transgene-free induced pluripotent mouse stem cells by the piggyBac transposon. Nat. Methods 6, 363–369. 10.1038/nmeth.1323 19337237 PMC2677165

[B132] Zhang S. C. Wernig M. Duncan I. D. Brüstle O. Thomson J. A. (2001). *In vitro* differentiation of transplantable neural precursors from human embryonic stem cells. Nat. Biotechnol. 19, 1129–1133. 10.1038/nbt1201-1129 11731781

[B133] Zhang W. Wang T. Pei Z. Miller D. S. Wu X. Block M. L.. (2005). Aggregated α-synuclein activates microglia: a process leading to disease progression in Parkinson's disease. FASEB J. 19, 533–542. 10.1096/fj.04-2751com 15791003

[B134] Zhou H. Wu S. Joo J. Y. Zhu S. Han D. W. Lin T.. (2009). Generation of induced pluripotent stem cells using recombinant proteins. Cell Stem Cell 4, 381–384. 10.1016/j.stem.2009.04.005 19398399 PMC10182564

[B135] Zhou W. Freed C. R. (2009). Adenoviral gene delivery can reprogram human fibroblasts to induced pluripotent stem cells. Stem Cells 27, 2667–2674. 10.1002/stem.201 19697349

